# An examination of relationships between vitamin B12 status and functional measures of peripheral neuropathy in young adult vegetarians

**DOI:** 10.3389/fnut.2023.1304134

**Published:** 2023-12-18

**Authors:** Taylor Arnold, Carol S. Johnston

**Affiliations:** Nutrition Program, College of Health Solutions, Arizona State University, Phoenix, AZ, United States

**Keywords:** vitamin B12, vegetarian, neuropathy, hand dexterity, balance, vibration sensitivity

## Abstract

**Introduction:**

Prevalence rates for vitamin B12 deficiency in U.S. adult vegetarians may exceed 30%, which is concerning given the role for this vitamin in numerous nervous system functions, including the synthesis of myelin sheaths. Defective myelin synthesis and repair are directly linked to peripheral neuropathy; yet, few investigations have examined how physical indicators of peripheral neuropathy (e.g., hand dexterity, vibration sensitivity and balance) are impacted in individuals adhering to vegetarian diets. This feasibility research explored the relationships between peripheral neuropathy and vitamin B12 status using a cross-sectional study design. In addition, a small pilot trial was conducted for limited-efficacy testing of vitamin B12 supplementation for reducing peripheral neuropathy.

**Methods:**

Healthy, able-bodied adults (*n* = 38; 19–40 years of age) reported exclusive adherence to a vegetarian or vegan diet for 3 years. Peripheral neuropathy was measured using a force plate for assessing balance, and a vibration sensitivity tester and pegboard tests to assess hand dexterity. Serum vitamin B12 and folate were measured using standard radioimmunoassay techniques.

**Results:**

Twenty-six percent of the sample displayed deficient or marginal vitamin B12 status (serum vitamin B12 <221 pmol/L). Participants with adequate vitamin B12 status scored 10% higher on the Purdue pegboard assembly test and 20% higher on the left hand adjusted functional dexterity test in comparison to participants with marginal-to-deficient vitamin B12 status (*p* < 0.05).

**Discussion:**

These data provide preliminary evidence that peripheral neuropathy can be detected in individuals with marginal-to-deficient vitamin B12 status.

## 1 Introduction

The prevalence of serum vitamin B12 deficiency has declined over the recent decades for U.S. adults >18 years of age, from 3.2% in the 1990’s to 1.8% in 2016 (1, 2); but for elderly populations (>50 years of age), serum vitamin B12 deficiency rates have trended higher: 4.8 and 5.8% for similar time periods (3, 4). Specific patient populations have even higher prevalence rates for B12 deficiency, including bariatric surgery patients (30%), those with malabsorptive diseases (6%–38%), and patients on certain medications such as antacids and metformin (14%–23%) (5–8). However, the prevalence rates for vitamin B12 deficiency in U.S. adult vegetarians appear markedly high, ranging from 30% to 47% (9). Data from the longitudinal EPIC-Oxford survey indicates that the prevalence of vitamin B12 deficiency may be as high as 52% among vegan adults in Europe (10).

Vitamin B12 is found in animal foods only (flesh foods, dairy, and eggs); hence, individuals who restrict or eliminate animal products from their diet are at risk for vitamin B12 deficiency. The high rates of vitamin B12 deficiency among vegetarian populations are concerning given the role for this vitamin in numerous nervous system functions, including the synthesis of neurotransmitters, nucleotides, and myelin sheaths as well as genomic and non-genomic methylation (11, 12). Untreated vitamin B12 deficiency may cause permanent neurological damage and can be fatal. Overt symptoms of vitamin B12 deficiency are often slow to develop and may not be detected by physicians until late in disease progression, as indicated by the name of the autoimmune disease caused by impaired vitamin B12 absorption: pernicious anemia. Vitamin B12 deficiency is commonly identified by secondary blood tests that are recommended when routine blood tests reveal macrocytic anemia. Serum vitamin B12 concentrations <148 pmol/L (200 pg./mL) indicate deficiency, and concentrations between 148 and 221 pmol/L indicate marginal status (13). However, neurologic symptoms can occur in the absence of anemia and even when serum vitamin B12 concentrations are within normal ranges (14).

Defective myelin synthesis and repair, which are characteristics of vitamin B12 insufficiency, are directly linked to peripheral neuropathy (15, 16). Yet, few investigations have examined how physical indicators of peripheral neuropathy are impacted in individuals at high risk for B12 deficiency. Physical indicators include manual hand dexterity (17), vibration sensitivity (18), and balance (19). These measures are widely used in research to assess clinical populations with neuropathy (e.g., diabetes mellitus, Parkinson’s disease, and rheumatoid arthritis), even in patients with low disease activity (20–25). But the usefulness of these indicators in non-clinical populations at risk for vitamin B12 deficiency has not been explored. Since these measures are considered sensitive indicators of neuropathy, and can be easily applied to reveal declines in neuronal processes, these measures could inform practitioners of pending health concerns in populations at high risk for vitamin B12 deficiency.

Young adult vegetarians are a suitable population to examine the subtle onset of neurological consequences of B12 deficiency, as they are more likely to have low or deficient vitamin B12 status than the average population; furthermore, the neurological consequences normally associated with aging and chronic disease conditions will likely not be present in this population. This feasibility research explored the utility of using hand dexterity, vibration sensitivity, and postural balance as indicators of vitamin B12 status using a cross-sectional study design. In addition, a small pilot trial was conducted for limited-efficacy testing of vitamin B12 supplementation. The research objective was to understand the relationships between deficient, marginal, and normal vitamin B12 status and peripheral neurological indicators in healthy adult vegetarians and vegans. Serum holo-transcobalamin II (TCII) and folate concentrations were also measured as these indicators can inform vitamin B12 assessments.

## 2 Methods

### 2.1 Participants

Vegetarian and vegan adults were recruited from a campus community through social media outreach, e-mail listservs, vegetarian support groups, and flyers posted on campus and at nearby vegan restaurants. Participants were 19–40 years of age and reported exclusive adherence to a vegetarian (devoid of all flesh foods) or vegan (devoid of all animal foods) diet for at least 3 years. Participants were healthy by self-report, e.g., not currently seeking treatment for unresolved or active disease conditions including neurological diseases, and not experiencing physical/mobility impairments. Exclusion criteria included an history of arthritis, carpel tunnel syndrome, hemophilia, and neurological movement disorders (i.e., Parkinson’s disease or multiple sclerosis), and pernicious anemia. Participants were excluded if they had a history of joint problems or joint replacement, severe vision problems, and/or false nails that extend past the fingertips. This study (both the cross-sectional trial and intervention trial) was approved by the Institutional Review Board at Arizona State University (STUDY00003188). All participants consented to the screening survey and provided formal written consent prior to study enrollment.

### 2.2 Cross-sectional study

Participant arrived at the testing facility after an overnight fast (no food or beverage for eight hours with the exception of water). Height and weight were measured using calibrated instruments, and a sample of blood was drawn. Blood was immediately processed, and extracted serum was frozen (−80°C) for later vitamin B12, transcobalamin II, and folate analyses. Participants were provided a small snack and completed health history and food frequency questionnaires. Following this short rest, participants engaged in four functional tests. For balance assessment, participants stood on the AMTI Accusway force plate (Advanced Mechanical Technology, Inc., Watertown, MA) with their feet at a 30° angle. They were instructed to stare at a fixed point on the wall while staying as still as possible for one minute. The force plate measured the center of pressure (COP) of the ground reaction force and the displacement in this force for one minute (e.g., the posture control sway to restore equilibrium) (26). COP displacement on the horizontal plane and the amplitude changes of COP in the anterior–posterior (AP) and medial-lateral (ML) phases were recorded as well as the COP path length and area (27). Lower scores on these measures indicate better postural stability.

The Vibratron II (Physitemp Instruments, LLC., Clifton, NJ) was used to measure cutaneous sensory loss (28). Using the pointer finger on their dominant hand, the participant was allowed 5 practice tests at the starting intensity to identify vibrations. When the vibrating rod was correctly identified, the vibration intensity for the next attempt was decreased by 10%. If a participant incorrectly identified the vibrating rod, that intensity was repeated. After two incorrect identifications, the intensity was increased by 10% and the test continued. If an incorrect identification was followed by a correct identification, one more attempt was made at that intensity. To eliminate the likelihood of a streak of correct guesses, all intensities below 0.7 vibration units (VU) were repeated twice. After five incorrect identifications, the test was completed. The vibration units of the five errors and the five lowest scores were compiled, with the highest and lowest of these numbers thrown out, and the vibration threshold in VU (or microns) was the average of the eight scores.

The Purdue Pegboard (Fabrication Enterprises, White Plains, NY) was used for assessing hand dexterity. The board has two parallel lines of holes down the center of the board with four wells at the top. The outside wells contain pins, the center right well contains washers, and the center left well contains sleeves. The Purdue Pegboard test (PPT) was administered in four parts: right hand, left hand, both hands, and assembly (29). The participant was allowed to practice with 3–4 pieces before the testing began. Participants were asked to place small metal pins into a line of holes in sequence, as quickly as possible in 30 s. Testing was conducted three times each with their right hand, left hand, and both hands. The score was the number of pins placed in the board. For the assembly test, participants created small towers with metal washers and sleeves, three times with both hands for 60 s each.

Functional Dexterity Test (FDT), which utilized a square pegboard with 16 holes and 16 pegs, was conducted to measure manual dexterity (30). The pegs had different colors on the top and bottom. The objective of the test was to turn all of the pegs over and place them back in their respective holes as quickly as possible. The test was conducted with each hand separately and scored by the number of seconds it took for the participant to complete turning each of the pegs over. Time penalties were added for any errors in completing the test (e.g., 5 s for each time the participant touched or rested on the board for support and 10 s if the participant dropped a peg). The total time without penalties was the net time and represents hand dexterity speed. The total time plus the penalties is the total time and represents the quality of the hand dexterity (30).

### 2.3 Pilot intervention

Participants in the cross-sectional study were invited to enroll in the pilot vitamin B12 supplementation trial. Participants signed a second consent form and were randomly assigned to the intervention group (1,000 μg vitamin B12 daily; Nature Made™) or to the control group (250 mg vinegar daily; Nature’s Life^™^). In a double-blind manner, each participant received an 8-week supply of pills (56 pills in opaque containers) and were instructed to consume one pill daily. Investigators were blinded to the contents of the bag. Participants also received a username and password for the Automated Self-Administered 24-Hour (ASA24^®^) Dietary Assessment Tool (National Cancer Institute, Bethesda, MD) to complete an online, 3-day diet record log. Participants were instructed to consume one pill each day for eight weeks. Throughout the 8-week trial, emails were sent to participants focusing on the importance of protocol adherence. An 8-week time line was selected based on the normalization of blood count when introducing B12 supplements to deficient patients (31, 32). The same assessments as conducted for the cross-sectional trial visit were repeated at the start of the intervention and at week eight.

### 2.4 Blood analyses

Serum vitamin B12 and folate were measured in duplicate using a commercial B12/Folate RIA kit (SimulTRAC-SNB Vitamin B12/Folate RIA Kit; MP Biomedicals; Santa Ana, CA) according to the manufacturer’s instructions. In a separate set of serum samples, holo-TCII was adsorbed by adding 100 μL of a slurry containing 3 mg silica to 500 μL serum, and the supernatant was assayed for B12 as outlined by Wickramasinghe and Fida (33). The value obtained represented holo-haptocorrin concentrations, and the holo-TCII concentration was determined by subtracting the holo-haptocorrin concentration from the total serum B12 concentration. Paired samples with high coefficients of variation (>10%) were re-run to reduce measurement error.

### 2.5 Statistical analysis

Published data were not available for peripheral neuropathy indicators in vitamin B12 deficiency; hence, a sample size of 30 was targeted as has been recommended for feasibility studies (34). As reference, in university students, dexterity training demonstrated a detectable difference of 1.96 for PPT assembly scores (SD = 1.26 and 1.98), suggesting a sample size of 24 (35). Statistical analysis was conducted using the Statistical Package for Social Sciences (IBM SPSS Statistics for Windows, Version 28.0. Armonk, NY: IBM Corp, Chicago IL). Normality was assessed, and nonparametric data were transformed as appropriate for testing. Data are reported as mean ± SD and *p* values ≤0.05 were considered significant. Spearman’s rank correlation was used to assess relationship between variables. Multivariate analysis was used to assess possible associations among variables, and univariate analyses were used to identify the significant interaction effects. Confounding variables were identified and controlled in the analyses. For the single instance where there was missing data, the cells were left blank and the sample size was reduced by one participant for the statistical testing.

## 3 Results

### 3.1 Cross-sectional study

A total of 351 individuals responded to the online screening survey, and 105 respondents qualified for the cross-sectional study. Many of the qualified respondents were unresponsive to follow-up or stated scheduling conflicts, and 38 qualified respondents were entered into the study (28.4 ± 5.5 years of age; 22.0 ± 2.7 kg/m^2^). A majority of participants were lacto-ovo vegetarians (60%), and 97% of participants had exclusively followed vegetarian/vegan diet plans for 3 years or more (Table 1). Participants were generally active and normal weight, and 84% of the sample were Caucasian. Fifty-five percent of participants took a supplement that contained vitamin B12, and 45% of participants reported taking other nutritional supplements regularly. None of the participants reported taking folic acid supplements. One participant reported regularly taking a medication that may impact vitamin B12 status (i.e., proton pump inhibitors). Although folate deficiency was not detected in the sample, 24% of the sample displayed deficient or marginal vitamin B12 status (serum vitamin B12 < 221 pmol/L) (Table 1). Three of 38 participants were left hand dominant, and two of these participants presented with adequate vitamin B12 status.

**Table 1:** Participant characteristics, diet adherence, and prevalence of nutrient deficiencies at study entry (*n* = 38).

	Mean ± SD	Min, max
Age, y	28.4 ± 5.5	19, 39
BMI, kg/m^2^	22.0 ± 2.7	17.3, 29.4
METS score*	62.6 ± 39.9	9, 166
Serum vitamin B12, pmol/L	352.8 ± 221.8	117.2, 1300.0
Serum transcobalamin II, pmol/L	55.5 ± 37.2	2.1, 170.1
Serum folate, nmol/L	42.2 ± 18.7	17.0, 98.6
Gender, M/F	9/29	
Lacto-ovo vegetarian, *n* (%)	23 (60)	
Vegan, *n* (%)	15 (40)	
Diet adherence ≥3 years, *n* (%)	37 (97)	
B12: deficient status (<148 pmol/L), *n* (%)	4 (11)	
B12: marginal status (148 to <221 pmol/L), *n* (%)	5 (13)	
Folate: deficient status (<6.7 nmol/L), *n* (%)	0	

*METS (Metabolic Equivalent of Task) score based on the Godin questionnaire; scores > 24 units indicate active lifestyle.

Balance indices and vibration threshold did not differ between the vitamin B12 status groups (Table 2). However, two balance measures, V(ML) Max and Area95, were weakly correlated with vitamin B12 status, *r* = −0.291 and − 0.294, respectively (*p* = 0.08). V(ML) Max is the maximum velocity for the anterior posterior sway peaks and Area95 is 95% of the total area formed by the COP trajectory for one minute of testing. The negative correlations suggest that lower vitamin B12 status is related to greater postural instability.

**Table 2:** Balance and vibration thresholds for participants in the cross-sectional study (*n* = 38).

	Serum vitamin B12 status	
	Low <221 pmol/L	High ≥221 pmol/L
	*n* = 9	*n* = 29
**Balance: COP parameters**		
V(AP) max, in/s	−3.9 ± 2.0	−3.0 ± 1.1
V(ML) max, in/s	3.3 ± 1.3	2.9 ± 1.3
V average, in/s	0.89 ± 0.36	0.96 ± 0.33
Path length, in	53.5 ± 21.4	57.3 ± 20.0
Area effective, in^2^	1.2 ± 1.3	1.2 ± 1.3
Area95, in^2^	2.8 ± 2.9	2.5 ± 2.1
**Vibration threshold**		
Vibration units	0.84 ± 0.20	0.83 ± 0.33
Microns	0.37 ± 0.18	0.40 ± 0.34

Data are mean ± SD, non-parametric scores were log-transformed prior to analyses. COP (center of pressure, a measure which fluctuates with posture control sway to restore equilibrium); V (velocity); AP (anterior–posterior); ML (medial-lateral); Area95 (95% of the total area formed by the COP path). Balance (lower scores indicate better posture stability): *p* = 0.345 for multivariate ANOVA controlling for sex. Vibration threshold (lower scores indicate greater sensitivity): *p* = 0.702 for multivariate ANOVA controlling for sex.

Scores for hand dexterity differed significantly between the vitamin B12 status groups as indicated by the MANOVA tests (*p* < 0.05), and the post-hoc tests revealed that participants with adequate vitamin B12 status scored more favorably on the Purdue pegboard assembly test (+10%) and the left hand adjusted functional dexterity test (+20%) in comparison to participants with marginal-to-deficient vitamin B12 status (Table 3). The MANOVA tests were repeated controlling for vitamin B12 supplementation, and the *p* values remained at or below 0.05 suggesting that the significant main effects remained. The assembly and left hand adjusted functional dexterity tests were also favorably correlated to vitamin B12 status (*r* = 0.374/*p* = 0.021 and *r* = −0.325/*p* = 0.046, respectively); no other scores correlated significantly with vitamin B12 status. Neither TCII or folate concentrations in serum were significantly correlated to any of the peripheral neuropathy measures.

**Table 3:** Hand dexterity measures for participants in the cross-sectional study (*n* = 38).

	Serum vitamin B12 status	
	Low <221 pmol/L	High ≥221 pmol/L
	*n* = 9	*n* = 29
**Purdue pegboard: fine dexterity**		
Right hand, pins/30 s	15.4 ± 1.7	15.4 ± 1.5
Left hand, pins/30 s	14.8 ± 1.6	14.4 ± 1.7
Both hands, pins/30 s	12.1 ± 1.8	12.5 ± 1.4
Right/left/both hands, pins/30 s	42.4 ± 4.3	42.4 ± 4.1
Assembly, pieces/60 s	27.0 ± 2.9	29.7 ± 4.0*
**Functional dexterity test**		
Right hand raw, seconds	20.2 ± 2.6	21.7 ± 4.7
Right hand adjusted, seconds	20.8 ± 2.9	23.7 ± 6.6
Left hand raw, seconds	25.1 ± 7.0	22.6 ± 5.5
Left hand adjusted, seconds	30.7 ± 8.8	25.2 ± 9.1**

Data are mean ± SD; non-parametric scores were log-transformed prior to analyses.

Fine Dexterity (higher scores indicate greater dexterity): *p* = 0.031 for multivariate ANOVA controlling for sex. *indicates weak trend for difference between groups, *p* = 0.05.

Functional Dexterity (lower scores indicate greater dexterity): *p* = 0.042 for multivariate ANOVA controlling for sex and age. **indicates weak trend for difference between groups, *p* = 0.097.

### 3.2 Pilot intervention

Twenty-four participants from the cross-sectional trial agreed to enter the 8-week intervention trial, and provided separate written consent. Six participants withdrew from the study after enrollment due to scheduling conflicts, and the remaining 18 participants completed the trial (vitamin B12 group: *n* = 10; control group: *n* = 8). Baseline characteristics did not differ by group (Table 4). Prior to study initiation, participants agreed to discontinue any vitamin B12 supplementation outside of that provided during the trial; one participant (vitamin B12 group) continued medication use during the trial. On average, participants in both groups reported 97% study compliance (e.g., daily ingestion of assigned pills). All participants in both groups were right hand dominant.

**Table 4:** Baseline characteristics, diet adherence, and prevalence of nutrient deficiencies for participants by intervention group: vitamin B12 (1,000 μg/d; *n* = 10) and control (250 mg/day vinegar; *n* = 8).

	Vitamin B12	Control
	Mean ± SD	
Age, y	26.0 ± 5.4	28.5 ± 5.8
BMI, kg/m^2^	22.0 ± 2.5	22.8 ± 3.4
METS score*	80.8 ± 50.6	60.0 ± 42.4
Serum vitamin B12, pmol/L	418.1 ± 339.4	336.0 ± 184.7
Serum transcobalamin II, pmol/L	57.5 ± 36.2	50.2 ± 35.9
Serum folate, nmol/L	42.2 ± 20.9	52.2 ± 22.8
Study adherence**, %	97.0 ± 4.1	96.7 ± 4.6
Gender, M/F	2/8	2/6
Lacto-ovo vegetarian, *n* (%)	7 (70%)	3 (38%)
Vegan, *n* (%)	3 (30%)	5 (63%)
Diet adherence ≥3 years, *n* (%)	10 (100%)	8 (100%)
B12: deficient status (<148 pmol/L), *n* (%)	0	0
B12: marginal status (148 to <221 pmol/L), *n* (%)	3 (30%)	1 (13%)
Folate: deficient status (<6.7 nmol/L), *n* (%)	0	0

*METS (metabolic equivalent of task) score based on the Godin questionnaire; scores >24 units indicate active lifestyle. **Percent of study days assigned pills were consumed (self-reported). There were no significant differences between groups (*p* > 0.05).

At the end of the 8-week intervention, the average vitamin B12 concentrations rose 154.8 ± 186.8 pmol/L in the vitamin B12 group (+37%) and fell 20.6 ± 137.9 pmol/L (−6%) in the control group (*p* = 0.042). At baseline, only three participants in the vitamin B12 group and one participant in the control group had marginal vitamin B12 status. At the completion of the trial, none of the participants in the vitamin B12 group fell in the marginal-to-deficient range for vitamin B12 status, but four participants in the control group had serum vitamin B12 concentrations <221 pmol/L. Average TCII concentrations rose 66.1 ± 126.6 pmol/L (+115%) in the vitamin B12 group in comparison to a reduction of 7.8 ± 65.4 pmol/L (−16%) in the control group (*p* = 0.115). For folate, serum averages fell in both groups: −5.0 ± 14.4 pmol/L (−12%) and − 14.3 ± 17.8 pmol/L (−27%) for the vitamin B12 and control groups, respectively (*p* = 0.239). Balance indices and vibration threshold did not differ between the vitamin B12 and control groups at baseline and the 8-week change data did not differ between groups (Table 5). Similarly, baseline data and the 8-week change data did not vary significantly between groups for any of the hand dexterity measures (Table 6).

**Table 5:** Baseline values and 8-week change data for balance and vibration threshold measures in the vitamin B12 (*n* = 10) and control (*n* = 7) groups.

	Vitamin B12	Control
**Balance: COP parameters**		
V(AP) Max, in/s	−2.47 ± 0.81	−2.88 ± 0.81
8-week change	0.03 ± 2.96	−0.94 ± 2.38
V(ML) Max, in/s	2.86 ± 1.54	2.54 ± 0.88
8-week change	−0.45 ± 3.23	1.08 ± 2.18
V average, in/s	0.82 ± 0.21	−0.94 ± 0.30
8-week change	−0.02 ± 0.65	0.13 ± 0.72
Path length, in	49.0 ± 12.5	56.5 ± 17.8
8-week change	−31.8 ± 14.3	−40.9 ± 18.7
Area effective, in^2^	1.37 ± 1.95	0.97 ± 0.51
8-week change	−1.19 ± 1.90	−0.70 ± 0.60
Area95, in^2^	2.51 ± 2.81	2.41 ± 1.43
8-week change	−2.09 ± 2.75	−1.91 ± 1.66
**Vibration threshold**		
Vibration units	0.92 ± 0.44	0.82 ± 0.19
8-week change	−0.12 ± 0.47	−0.12 ± 0.18
Microns	0.51 ± 0.51	0.35 ± 0.15
8-week change	−0.17 ± 0.49	−0.10 ± 0.15

COP (center of pressure, a measure which fluctuates with posture control sway to restore equilibrium); V (velocity); AP (anterior–posterior); ML (medial-lateral); Area95 (95% of the total area formed by the COP path). Balance (lower scores indicate better posture stability); Vibration threshold (lower scores indicate greater sensitivity). COP parameters: *p* = 0.0.676 for multivariate ANOVA controlling for sex. Vibration threshold: *p* = 0.679 for multivariate ANOVA controlling for sex. One control participant had missing data reducing the control sample size to seven. Baseline values were not different between groups.

**Table 6:** Baseline values and 8-week change data for hand dexterity measures in the vitamin B12 (*n* = 10) and control (*n* = 8) groups.

	Vitamin B12 *n* = 10	Control *n* = 8
**Purdue pegboard: fine dexterity**		
Right hand, pins/30 s	15.4 ± 1.2	165.9 ± 1.6
8-week change	0.80 ± 1.10	0.46 ± 0/73
Left hand, pins/30 s	14.8 ± 1.0	14.3 ± 2.1
8-week change	0.10 ± 0.67	0.67 ± 0.59
Both hands, pins/30 s	12.7 ± 1.2	12.3 ± 1.7
8-week change	0.20 ± 0.72	0.08 ± 0.71
Right/left/both hands, pins/30 s	42.9 ± 3.2	42.5 ± 4.9
8-week change	1.10 ± 1.72	1.21 ± 1.39
Assembly, pieces/60 s	29.5 ± 2.9	28.4 ± 5.7
8-week change	1.50 ± 2.50	1.21 ± 4.10
**Functional dexterity test**		
Right hand raw, seconds	21.8 ± 3.1	23.4 ± 8.6
8-week change	−0.07 ± 3.12	−1.12 ± 3.76
Right hand Adjusted, seconds	21.8 ± 3.1	23.4 ± 8.6
8-week change	2.43 ± 5.19	0.13 ± 10.3
Left hand raw, seconds	22.8 ± 5.2	24.5 ± 6.9
8-week change	−0.85 ± 3.32	0.04 ± 2.23
Let hand adjusted, seconds	25.8 ± 6.4	29.5 ± 12.6
8-week change	−2.85 ± 4.88	−2.95 ± 6.09

Data are mean ± SD. Fine dexterity (higher scores indicate greater dexterity). Functional dexterity (lower scores indicate greater dexterity). Fine dexterity: *p* = 0.0.325 for multivariate ANOVA controlling for sex and age. Functional dexterity: *p* = 0.839 for multivariate ANOVA controlling for sex and age. Baseline values were not different between groups.

## 4 Discussion

In the cross-sectional trial, vitamin B12 status was significantly correlated to several hand dexterity measures. Furthermore, participants with adequate vitamin B12 status scored 10–20% higher on left-hand functional dexterity and the Purdue Pegboard assembly task than the participants with deficient-to-marginal vitamin B12 status. Although not previously reported in the literature, reduced manual dexterity could be anticipated in individuals with vitamin B12 deficiency since the condition results in defective myelin synthesis leading to nervous system disfunctions including peripheral neuropathy. Participants were young adults who adhered strictly to vegetarian diets and were healthy by self-report. Based on normative scores for the Purdue Pegboard Test, participant scores averaged in the standard range for the hand dexterity measures with the exception that the deficient-to-marginal vitamin B12 group scored below standard for the assembly measure (36). This reduced score was in the range reported for patients with diabetic mononeuropathy and polyneuropathy of their hands (37).

Balance and postural stability are also indicators of peripheral neuropathy. Although significant differences on averaged balance measures were not detected between vitamin B12 adequate participants and those with deficient-to-marginal vitamin B12 status, weak negative correlations were noted for two indices and vitamin B12 nutriture: V(ML) Max, the maximum velocity for the anterior–posterior sway peaks, and Area95, 95% of the total area formed by the COP trajectory for one minute of testing (*p* = 0.08). Greater sway and COP trajectory in participants with lower vitamin B12 concentrations indicate the possibility of reduced postural balance in these individuals.

Reduced hand functionality and posture unsteadiness can decrease quality of life by negatively impacting daily activities or work performance. In patients with diabetes, hand dexterity scores correlated with quality of life measures, linking the ability to effectively accomplish manual tasks, such as picking up small objects, typing, and writing, with satisfaction and accomplishment (37). Keramiotou et al. (22) demonstrated a strong association between hand dexterity, performance of daily activities, and quality of life in patients with systemic lupus erythematosus. Similarly, Wong-Yu et al. (38) noted correlations between hand dexterity measures and quality of life in patients with Parkinson’s disease. Both manual dexterity and grip strength were reduced 20%–40% in patients compared to healthy controls, yet only the manual dexterity measures were related to the quality of life measures. Llamas-Velasco et al. (39) demonstrated that a 24-week home calligraphic training program signifyingly improved hand dexterity and quality of life in patients with Parkinson’s disease, further supporting a strong link between manual dexterity and wellbeing.

Recent evidence suggests that hand dexterity relies on the cognitive processes that characterize executive function, the higher-level cognitive skills that coordinate mental capacity (40). Koch et al. found that 5-Hz repetitive transcranial magnetic stimulation (rTMS) over the scalp site corresponding to the primary motor cortex, a component of the frontal lobe region housing executive function, improved hand dexterity as reflected by a reduction of the time required to complete the pegboard task in patients with multiple sclerosis (41). Moreover, recent research in older adults demonstrated that reduced hand dexterity was linked to increased risk of cognitive impairment (42). The requirement for vitamin B12 in neuronal myelination to maintain peripheral and central nerves as well as motor fibers supports a relationship between vitamin B12 status and executive function (43). Hence, the possibility that deficient-to-marginal vitamin B12 status may manifest as subtle cognitive impairment as well as peripheral neuropathy deserves investigation (44).

This study had a number of limitations. As a feasibility study, power was not calculated, and the sample size was small and unrepresentative. We did not exclude participants supplementing vitamin B12 for the cross-sectional trial, and, for the intervention trial, we did not enact a washout period for the participants who discontinued use of vitamin B12 supplements prior to the start of the trial. The procedures used to test peripheral neuropathy (e.g., hand dexterity, vibration sensitivity, and postural balance) have been reported to have learning effects, but for the cross-sectional trial, there was a one-time administration of the tests, which has been reported as reliable and valid in a patient population (45). Study strengths include that the intervention was double-blinded and that self-reported compliance was 97%.

## 5 Conclusion

Serum vitamin B12 is not a definitive marker for vitamin deficiency as clinical symptoms of deficiency can occur above the standard cutoffs (31). Although it is well recognized that peripheral neuropathy is a classic symptom of vitamin B12 deficiency, physical indicators for peripheral neuropathy, such as hand dexterity and balance, have not been utilized as a measure of disease progression in this population. These data provide preliminary evidence that peripheral neuropathy can be detected in individuals with marginal-to-deficient vitamin B12 status. Future investigations are warranted to describe the extent and progression of peripheral neuropathy in larger samples of individuals with marginalized vitamin B12 status.

## Data availability statement

The raw data supporting the conclusions of this article will be made available by the authors, without undue reservation.

## Ethics statement

The studies involving humans were approved by the Institutional Review Board, Arizona State University. The studies were conducted in accordance with the local legislation and institutional requirements. The participants provided their written informed consent to participate in this study.

## Author contributions

TA: Conceptualization, Formal analysis, Methodology, Writing – review & editing, Funding acquisition, Investigation, Project administration. CJ: Conceptualization, Formal analysis, Methodology, Writing – review & editing, Supervision, Writing – original draft.
